# Correlation between the structure and skin permeability of compounds

**DOI:** 10.1038/s41598-021-89587-5

**Published:** 2021-05-12

**Authors:** Ruolan Zeng, Jiyong Deng, Limin Dang, Xinliang Yu

**Affiliations:** Hunan Provincial Key Laboratory of Environmental Catalysis & Waste Regeneration, College of Materials and Chemical Engineering, Hunan Institute of Engineering, Xiangtan, 411104 Hunan China

**Keywords:** Environmental sciences, Chemistry

## Abstract

A three-descriptor quantitative structure–activity/toxicity relationship (QSAR/QSTR) model was developed for the skin permeability of a sufficiently large data set consisting of 274 compounds, by applying support vector machine (SVM) together with genetic algorithm. The optimal SVM model possesses the coefficient of determination *R*^2^ of 0.946 and root mean square (*rms*) error of 0.253 for the training set of 139 compounds; and a *R*^2^ of 0.872 and *rms* of 0.302 for the test set of 135 compounds. Compared with other models reported in the literature, our SVM model shows better statistical performance in a model that deals with more samples in the test set. Therefore, applying a SVM algorithm to develop a nonlinear QSAR model for skin permeability was achieved.

## Introduction

Modeling the penetration of manmade and naturally derived chemicals through human skin is of great importance for pharmaceutical and cosmetic industries, as well as toxicology and risk assessment of environmental and occupational hazards. It is very time-consuming and expensive to estimate the skin permeability of chemicals. Further, there are many ethical challenges associated with human and animal testing for assessment of skin permeability^1,2^.

Quantitative structure–activity/toxicity relationship (QSAR/QSTR) models^3–6^ can be used for the prediction of physicochemical property of compounds, even for those that have not been synthesized. Some researchers have carried out QSAR studies for skin permeability of chemicals (the logarithm of the skin permeability coefficients, log *K*_p_).

Patel et al. developed QSAR models for the skin permeability of 158 chemicals with multiple linear regression (MLR) analysis^7^. The model based on four descriptors has an excellent fit to the data with a coefficient of determination of *R*^2^ of = 0.90. Fujiwara et al. proposed MLR QSARs for the skin permeability of 94 structurally diverse compounds^8^. The models obtained from ten data sets of the skin permeability possess high *R*^2^ values with an average *R*^2^ of 0.815. Magnusson et al. introduced a regression model (*R*^2^ = 0.760) for the skin permeability of 269 compounds^9^. They found that molecular weight was the main determinant of log *K*_P_ and QSAR model can be improved when other descriptors such as melting point and hydrogen bonding acceptor capability were added. Chauhan and Shakya built a QSAR model for the skin permeability from the training set of 150 compounds through partial least-squares regression^10^. The model with a *R*^2^ of 0.936 for the training set was validated by the test set of 53 compounds. The root mean square (*rms*) error and *R*^2^ from the test set were equal to 0.670 and 0.542. Xu et al. proposed an expanded version of a linear free-energy relationship model for the skin permeability of complex chemical mixtures^11^. The model (*R*^2^ = 0.70) showed a better fit and predictive power compared with the simple model (*R*^2^ = 0.21). Chen et al. generated a MLR model for the skin permeability with four molecular descriptors^12^. The model has a *R*^2^ of 0.858 for the training set (85 compounds), and 0.839 for the test set (21 compounds), which are accurate and acceptable. All these QSAR models referred to were obtained with the linear techniques.

Generally, nonlinear QSAR models possess better statistical performance than linear QSAR models because of the nonlinear correlation between molecular physicochemical properties and structure descriptors. Neely et al. constructed a nonlinear artificial neural network (ANN) model for the skin permeability of 160 molecular structures^13^. The ANN model (10-3-7-1) based on ten descriptor and two hidden layers had an absolute-average percentage deviation, *rms* error, and *R* of 8.0%, 0.34, and 0.93, respectively. Khajeh and Modarress introduced a novel nonlinear QSAR model for the skin permeability of 283 compounds with the hybrid of ANN and a fuzzy inference system, adaptive neuro-fuzzy inference system (ANFIS)^14^. The ANFIS model was based on a training set of 225 compounds and validated by a test set of 58 compounds. The *R*^2^ values for the two sets were 0.899 and 0.890, respectively. The model possesses good predictive ability, although there are nine compounds in duplicate in the data set.

ANN algorithm may easily fall into a local minimum value and possesses the disadvantages of slow convergence speed^15^. Support vector machine (SVM) algorithm is based on the principle of structural risk minimization. SVMs can effectively avoid local optimums and have unique advantages in solving practical problems such as limited training samples, high dimensional and nonlinear data. The aim of this study was to develop a nonlinear SVM QSAR model for the skin permeability of a sufficiently large data set consisting of 274 compounds.

## Materials and methods

Khajeh and Modarress reported 283 compounds and their experimental log *K*_p_ values^14^. After careful investigation, we found that the sample, p-Chlorobenzene, should be 1-chloro-4-nitrobenzene and 4-Chloro-4-phenylenediamine should be 4-Chloro-m-phenylenediamine. There are no counterions or organometallics in the data set. The molecular weights of 283 compounds were calculated with ChemDraw Ultra 8.0 in ChemOffice 2004. These molecules possessing the same molecular weights were checked carefully to identify the duplicates. There are nine compounds in duplicate, including 4-phenylenediamine (1,4-benzenediamine), 4-hydroxynitrobenzene (4-nitrophenol), methylhydroxybenzoate (methyl 4-hydroxybenzoate), 1,2-benzenediamine (2-phenylenediamine), 2-naphthol (naphthalene-2-ol), 2-nitro-1,4-phenylenediamine (2-nitro-4-phenylenediamine), 1-nonanol (Nonanol), 4-chloro-1,3-phenylenediamine (4-Chloro-m-phenylenediamine), and 1-heptanol (Heptanol). After these duplicates were deleted, 274 compounds were obtained. Table S1 in “Supplementary Materials” shows their SMILES structures and the log *K*_p_ values. The units for skin permeability coefficients *K*_p_ are cm/h and these log *K*_p_ values ranged from − 6.10 to − 0.76. The Kennard-Stone algorithm^16^ was used to group the compounds in the training set (139 compounds) and test set (135 compounds). The training set was used to adjust model parameters and train QSAR models; and the test set was used to validate the models.

ChemDraw Ultra 8.0 in ChemOffice 2004 was adopted to generate the structures of 274 compounds, which were converted into three-dimensional structures with Chem3D Ultra 8.0 and optimized with a semi-empirical AM1 method in MOPAC. Dragon 6.0^17^ was used to calculate 4885 molecular descriptors for each compound. After some molecular descriptors that equal a constant or their correlation coefficients are above 0.90 were deleted, 1820 descriptors (including Neoplastic-80) were obtained for descriptor selection. Stepwise MLR analysis in IBM SPSS Statistical 19 was performed to select the optimal subset of descriptors and develop MLR models.

For non-linear regression, SVM algorithms map input variables into high-dimensional feature space, from which linear regression analysis is carried out^18,19^. For sample data, $\left( {y_{1} ,x_{1} } \right), \ldots ,\left( {y_{l} ,x_{l} } \right),\quad x \in R^{n} ,\;y \in R$, the regression function is expressed as follows:

1 $$ f(x) = \sum\limits_{i}^{n} {\varphi (x_{i} )w + b} $$

The optimal regression function can be obtained by means of the following minimization problem:

2 $$ \mathop {min}\limits_{{w,b,\xi ,\xi^{*} }} J(w,\upxi ,\upxi ^{*} ,b) = \frac{1}{2}\left\| {\left. w \right\|} \right.^{2} + C\sum\limits_{i} {(\xi_{i} + \xi_{i}^{*} )} $$

Subject to Eqs. (3–4):

3 $$ y_{i} - \varphi^{T} (x_{i} )w - b \le \varepsilon + \xi_{i} $$

4 $$ \varphi^{T} (x_{i} )w + b - y_{i} \le \varepsilon + \xi_{i}^{*} $$

In SVM regression, the *ε*-insensitive loss function is employed for minimizing the training error:

5 $$ \left| {f\left( x \right) - y} \right|_{\varepsilon } = \left\{ {\begin{array}{*{20}l} {0,} \hfill & {\left| {f\left( x \right) - y} \right| < \varepsilon } \hfill \\ {\left| {f\left( x \right) - y} \right| - \varepsilon ,} \hfill & {\left| {f\left( x \right) - y} \right| \ge \varepsilon } \hfill \\ \end{array} } \right. $$

Thus, Eq. (1) is:

6 $$ f{(}x{)} = \sum\limits_{i}^{n} {{(}a_{i} - a_{i}^{*} {)}} \varphi {(}x_{i} {)} \cdot \varphi {(}x{)} + b $$

By applying a kernel function *k*(*x, y*), Eq. (6) can be expressed as:

7 $$ f{(}x{)} = \sum\limits_{i}^{s} {{(}a_{i} - a_{i}^{*} {)}} K{(}x{,}y{)} + b $$

Gaussian radial basis function (RBF) was used in this work:

8 $$ K{\text{(X}}_{i} {\text{,X}}_{j} {)} = {\text{exp}}\left( { - \gamma \left\| {{\text{X}}_{i} - {\text{X}}_{j} } \right\|^{2} } \right) $$

For SVM models, their SVM parameters *C* and *γ* can affect greatly their prediction performance. Both *C* and *γ* were optimized with the genetic algorithm. In this study, the LibSVM toolbox^20^ working on Matlab platform was used to develop models, which can be downloaded freely from https://www.csie.ntu.edu.tw/~cjlin/libsvm/.

## Results and discussion

After carrying out stepwise MLR analysis in IBM SPSS Statistical 19 for the skin permeability log *K*_p_ of 274 compounds and 1820 descriptors, a three-descriptor QSAR model was obtained, which includes A log P, X3v, and Neoplastic-80.

**Figure 1:**
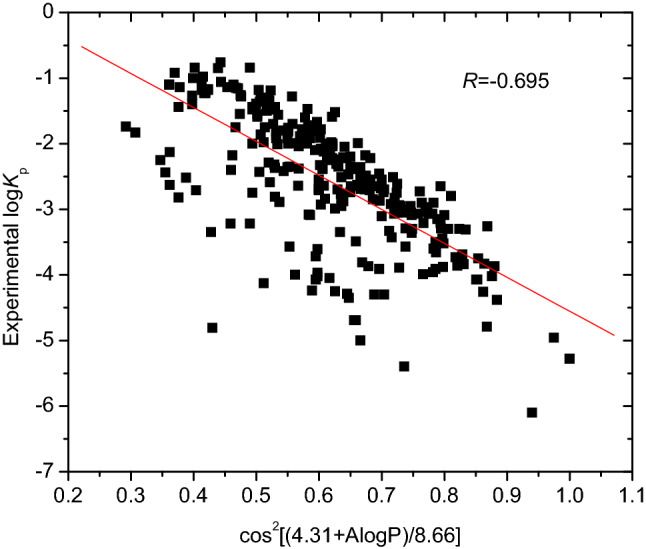
Plot of the descriptor cos^2^[(4.31 + A log P)/8.66] versus log *K*_p_, generated by OriginPro 7.5 SR1.

The Ghose–Crippen–Viswanadhan octanol–water partition coefficient (A log P) is based on the A log P model^21^ and calculated by:

9 $$ {\text{A}}\log {\text{P}} = \sum\limits_{i} {n_{i} a_{i} } $$

where *n*_*i*_ is the number of atom of type *i* and *a*_*i*_ is the corresponding hydrophobicity constant. Previous works have shown that A log P is positively correlation with skin permeability log *K*_p_. In this work, the descriptors were converted to a new descriptor cos^2^[(4.31 + A log P)/8.66]. An analysis of cos^2^[(4.31 + A log P)/8.66] with respect to the skin permeability log *K*_p_ of 274 compounds resulted in regression Eq. (10) and statistical parameters:

10 $$ \begin{aligned} & {\text{log}}\;K_{{\text{p}}} = 0.{624}{-}{5}.{\text{178 cos}}^{{2}} \left[ {\left( {{4}.{31} + {\text{A}}\;{\text{log}}\;{\text{P}}} \right)/{8}.{66}} \right] \\ & n = {274},\;R = 0.{695},\;R^{{2}} = 0.{483},\;R^{{2}}_{{{\text{adj}}}} = 0.{481},\;{\text{se}} = 0.{713},\;F = {253}.{9}0{3} \\ \end{aligned} $$

where *n* is the number of samples in the training set, *R*^2^ is the coefficient of determination, *R*^2^_adj_ is the adjusted *R* square, *se* is the standard error of the estimate, and *F* is the Fischer ratio. Figure 1 shows the correlation between cos^2^[(4.31 + A log P)/8.66] and log *K*_p_. The descriptor cos^2^[(4.31 + A log P)/8.66] (or A log P) describes the hydrophobic character of a compound and is related to log *K*_p_.

Connectivity indices are used widely in QSARs. They are based on the H-depleted molecular graph whose vertexes belong to non-hydrogen atom and are correlated with the number of connected non-hydrogen atoms^17^. The general formula for calculating connectivity indices is:

11 $$ Xk = \sum\limits_{j = 1}^{k} {\left( {\prod\limits_{i = 1}^{n} {\delta_{i} } } \right)}^{{{{ - 1} \mathord{\left/ {\vphantom {{ - 1} 2}} \right. \kern-\nulldelimiterspace} 2}}} $$

where *n* is the number of vertices; *k* is an integer ranging from 0 to 5, denoting the total number of *k*th order paths present in the molecular graph; and *δ* is the vertex degrees. Valence connectivity indices (*Xk*v) can be used to account for the presence of heteroatoms in the molecule as well as of double and triple bonds, by means of replacing the vertex degree with the valence vertex degree. The valence connectivity index of order 3, X3v, describes molecular size and shape.

By correlating log *K*_p_ to the two descriptors, cos^2^[(4.31 + A log P)/8.66] and X3v, we obtained the following regression equation:

12 $$ \begin{aligned} & {\text{log}}\;K_{{\text{p}}} = {2}.{23}0{-}{6}.{\text{643 cos}}^{{2}} \left[ {\left( {{4}.{31} + {\text{A}}\;{\text{log}}\;{\text{P}}} \right)/{8}.{66}} \right]{-}0.{\text{245 X3v}} \\ & n = {274},\;R = 0.{918},\;R^{{2}} = 0.{844},\;R^{{2}}_{{{\text{adj}}}} = 0.{842},\;{\text{se}} = 0.{393},\;F = {731}.0{89} \\ \end{aligned} $$

**Figure 2:**
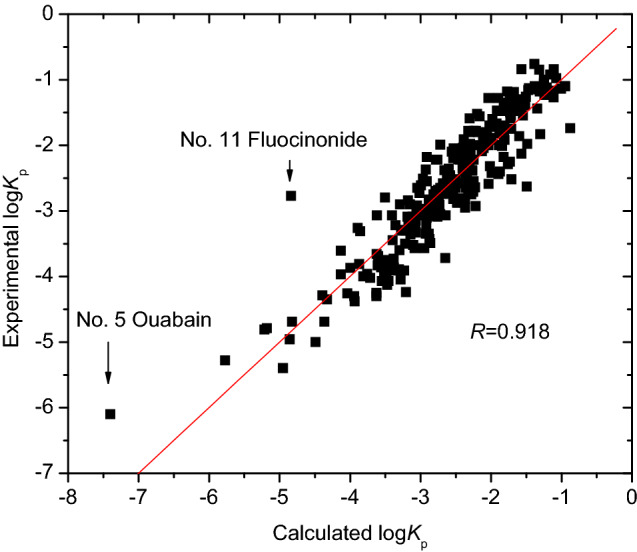
Plot of experimental versus calculated log *K*_p_ with Eq. (12), generated by OriginPro 7.5 SR1.

Compared with Eq. (10), the quality of Eq. (12) improved noticeably when the descriptor X3v was added. Figure 2 shows the correlation between the experimental and calculated log *K*_p_ with Eq. (12). As illustrated in Fig. 2, there were two samples, ouabain (No. 5 in Table S1), and fluocinonide (No. 11) with larger prediction errors for log *K*_p_. Thus, more molecular descriptors should be added.

The descriptor Ghose–Viswanadhan–Wendoloski antineoplastic-like index at the qualifying range that covers approximately 80% of the drugs studied, Neoplastic-80, depends on A log P and reflects molecular polarity and hydrophobicity^17^. The Neoplastic-80 value of a molecule that has a benzene ring, heterocyclic ring, aliphatic amine, carboxamide group, alcoholic hydroxyl group, carboxy ester and/or keto group, was equal to 1, when its A log P value is in the range of − 1.5 to 4.7, the molar refractivity of 43–128, the molecular weight of 180–470, and the total number of atoms of 21–63; otherwise Neoplastic-80 equals zero. A molecule with larger Neoplastic-80 might have a smaller log *K*_p_ value. Carrying out regression analysis between log *K*_p_ of 274 compounds and the three descriptors stated above resulted in Eq. (13):

13 $$ \begin{aligned} & {\text{log}}\;K_{{\text{p}}} = {2}.{2}0{9}{-}{6}.{\text{698 cos}}^{{2}} \left[ {\left( {{4}.{31} + {\text{A}}\;{\text{log}}\;{\text{P}}} \right)/{8}.{66}} \right]{-}0.{\text{174 X3v}}{-}0.{7}0{\text{4 Neoplastic}} - {8}0 \\ & n = {274},\;R = 0.{945},\;R^{{2}} = 0.{893},\;R^{{2}}_{{{\text{adj}}}} = 0.{892},\;{\text{se}} = 0.{324},\;F = {756}.{879} \\ \end{aligned} $$

**Figure 3:**
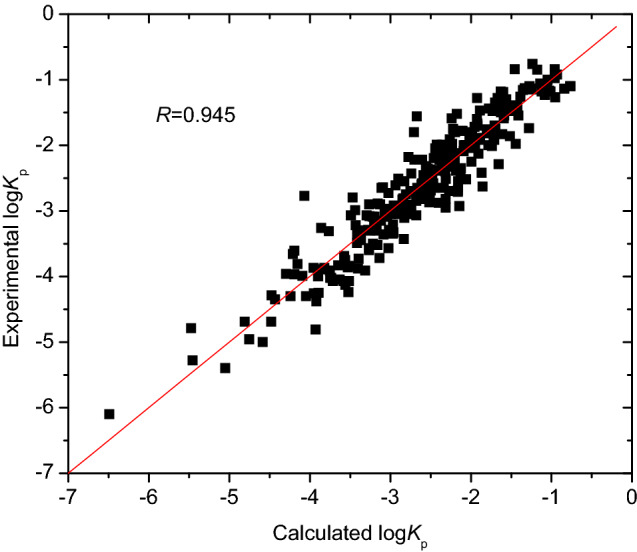
Plot of experimental versus calculated log *K*_p_ with Eq. (13), generated by OriginPro 7.5 SR1.

The correlation coefficient *R* of 0.945 in Eq. (13) was slightly higher than the 0.942 of the model^13^. Moreover, Eq. (13) has accurate prediction for the skin permeability log *K*_p_ of compounds including the two samples (Nos. 5 and 11 in Table S1 in “Supplementary Materials”) stated above, since Fig. 3 shows that there are no samples with obvious larger errors. When the descriptor A log P, together with X3v and Neoplastic-80, was directly used to develop the MLR model, its correlation coefficient *R* was only 0.939, which was lower than the 0.945 of Eq. (13). Thus the three descriptors, cos^2^[(4.31 + A log P)/8.66], X3v, and Neoplastic-80 shown in Table S1 in “Supplementary Materials” were used to develop QSAR models.

A correlation analysis between the skin permeability log *K*_p_ of 139 compounds in the training set and the three descriptors resulted in Eq. (14) (i.e., MLR model):

14 $$ \begin{aligned} & {\text{log}}\;K_{{\text{p}}} = {2}.0{68}{-}{6}.{\text{515 cos}}^{{2}} \left[ {\left( {{4}.{31} + {\text{A}}\;{\text{log}}\;{\text{P}}} \right)/{8}.{66}} \right]{-}0.{\text{722 X3v}}{-}0.{\text{168 Neoplastic}} - {8}0 \\ & n = {139},\;R = 0.{949},\;R^{{2}} = 0.{9}0{1},\;R^{{2}}_{{{\text{adj}}}} = 0.{899},\;{\text{se}} = 0.{349},\;F = {41}0.{148} \\ \end{aligned} $$

The characteristics of molecular descriptors in MLR model are listed in Table 1. As can been observed in Table 1, the three descriptors, cos^2^[(4.31 + A log P)/8.66], X3v, and Neoplastic-80 descriptor all were significant and made a contribution to log *K*_p_, because their significance values (or *P* values) are less than 0.05. In addition, their variance inflation factors (VIF) were far less than ten suggesting that the three descriptors describe different structure factors affecting skin permeability log *K*_p_. The *t*-test can be used to measure the significance of descriptors in making a contribution to molecular physicochemical properties. The higher the absolute value of the *t*-test, the greater the significance of the descriptor. According to the *t*-test values in Table 1, the absolute values of *t*-test increased in the sequence: Neoplastic-80, X3v, and cos^2^[(4.31 + A log P)/8.66], the significance of descriptors increased in the same sequence.

**Table 1:** Characteristics of molecular descriptors in MLR model.

Descriptor	Coefficients	Std. error	*t*-test	*P-*value	VIF
Constant	2.068	0.145	14.221	0.000	–
cos^2^[(4.31 + A log P)/8.66]	− 6.515	0.206	− 31.625	0.000	1.102
X3v	− 0.722	0.074	− 9.750	0.000	1.420
Neoplastic-80	− 0.168	0.012	− 14.248	0.000	1.442

**Figure 4:**
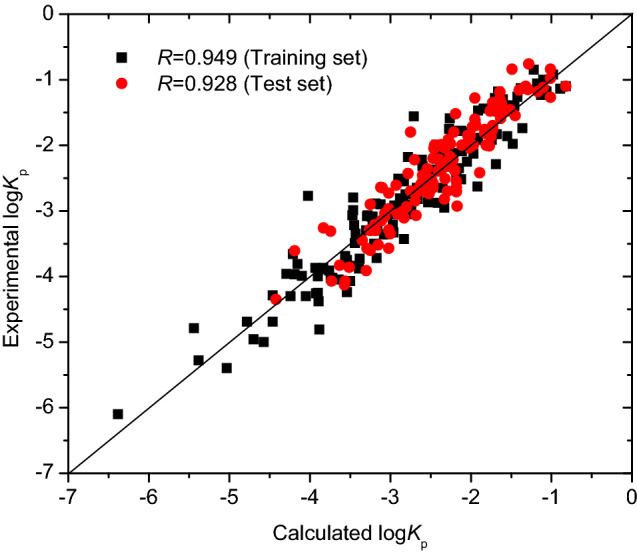
Plot of experimental versus predicted log *K*_p_ with Eq. (14), generated by OriginPro 7.5 SR1.

The MLR model was further used to predict the skin permeability log *K*_p_ of 135 compounds in the test set. The correlation coefficient *R* of the test set was 0.928. The *rms* errors for the training set, test set and total set were 0.343, 0.302, and 0.323, respectively. The prediction log *K*_p_ values are illustrated in Fig. 4 and listed in Table S1 in “Supplementary Materials”.

The three molecular descriptors used in Eq. (14) were used as input variables to develop SVM models for skin permeability log *K*_p_ from the training set of 139 compounds, by applying the LibSVM toolbox in the MATLAB R2014a software platform. A genetic algorithm was adopted to optimize the SVM parameters *C* and *γ* under the following conditions: the searching range of parameters *C* was [0, 1000], the searching range of *γ* was [0, 10], the *m* in *m*-fold-cross-validation was 5, the maximum generation was 200, the maximum population size was 20, and the *ε* in the *ε*-insensitive loss function was 0.001.

**Figure 5:**
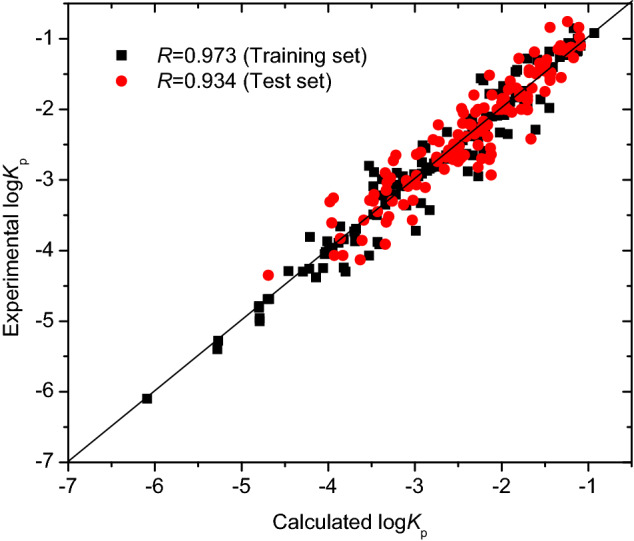
Plot of experimental versus predicted log *K*_p_ with SVM model, generated by OriginPro 7.5 SR1.

The optimization results for the SVM model were obtained: the parameters *C* being 7.2906 and *γ* being 1.7200, and the internal correlation coefficient based on leave-one-out (LOO) cross-validation method being 0.82. The optimal SVM model was further validated with the test set of 135 compounds. The SVM prediction results are listed in Table S1 in “Supplementary Materials” and illustrated in Fig. 5. The coefficient of determination *R*^2^ and *rms* error for the training set of 139 compounds were 0.946 and 0.253, respectively; *R*^2^ and *rms* for the test set of 135 compounds were 0.872 and 0.302, respectively; and *R*^2^ and *rms* error for the total set were 0.925 and 0.270, respectively. The *rms* errors of 0.253, 0.302, and 0.270, respectively, for the training set, test set and total set from the SVM model were lower than those (0.343, 0.302, and 0.323, respectively) of Eq. (14) (MLR model) in this study. Therefore, there were non-linear relationships between the skin permeability log *K*_p_ and molecular descriptors used.

The SVM model was further evaluated with the criteria by Golbraikh and Tropsha:^22^

15 $$ q_{{{\text{ext}}}}^{{2}} = 1 - \frac{{\sum {\left( {y_{i} - \widetilde{y}_{i} } \right)^{2} } }}{{\sum {\left( {y_{i} - \overline{y}_{{{\text{train}}}} } \right)^{2} } }} = 0.905 > 0.5 $$

16 $$ 0.85 < k = \frac{{\sum {y_{i} \widetilde{y}_{i} } }}{{\sum {\widetilde{y}_{i}^{2} } }} = 1.001 < 1.15 $$

17 $$ 0.85 < k^{\prime} = \frac{{\sum {y_{i} \widetilde{y}_{i} } }}{{\sum {y_{i}^{2} } }} = 0.985 < 1.15 $$

18 $$ R_{0}^{2} = 1 - \frac{{\sum {\left( {\widetilde{y}_{i} - y_{i}^{{r_{0} }} } \right)^{2} } }}{{\sum {\left( {\widetilde{y}_{i} - \overline{{\widetilde{y}}} } \right)^{2} } }} = 0.858 $$

19 $$ R_{0}^{\prime 2} = 1 - \frac{{\sum {\left( {y_{i} - \widetilde{y}_{i}^{{r_{0} }} } \right)^{2} } }}{{\sum {\left( {y_{i} - \overline{y} } \right)^{2} } }} = 0.872 $$

20 $$ {{\left| {R^{2} - R_{0}^{2} } \right|} \mathord{\left/ {\vphantom {{\left| {R^{2} - R_{0}^{2} } \right|} {R^{2} }}} \right. \kern-\nulldelimiterspace} {R^{2} }} = 0.016 < 0.1; $$

21 $$ {{\left| {R^{2} - R_{0}^{\prime 2} } \right|} \mathord{\left/ {\vphantom {{\left| {R^{2} - R_{0}^{\prime 2} } \right|} {R^{2} }}} \right. \kern-\nulldelimiterspace} {R^{2} }} = 0 < 0.1 $$

where $q_{ext}^{2}$ is external correlation coefficient; *R*_0_^2^ and *R*_0_^′2^ are determination coefficients of the predicted vs. the observed values and of the observed vs. the predicted values, respectively; *k* and *k*′ are slopes of regression lines of the predicted vs. the observed values and of the observed values vs. the predicted values; $\overline{y}_{{{\text{train}}}}$ is the average value of the training set; y_*i*_ and $\overline{y}_{i}$ are the observed and the predicted activities, respectively; $y_{i}^{{r_{0} }} = k\widetilde{y}_{i}$ and $\widetilde{y}_{i}^{{r_{0} }} = k^{\prime}y_{i}$. Obviously, our SVM model satisfied the validation criteria^22,23^.

The coefficient of determination *R*^2^ (= 0.946) in this study is higher than the *R*^2^ of 0.90^7^, 0.815^8^, 0.760^9^, 0.936^10^, 0.70^11^, 0.858^12^, and 0.93^13^. In addition, the *rms* errors of the training set, test set and total set from the ANFIS model of Khajeh and Modarress that dealt with the 283 samples were 0.318, 0.308, and 0.316 respectively^14^, which were greater than the *rms* errors ( 0.253, 0.302, and 0.270, respectively) from our SVM model. Compared with results of other models reported in the literature^9–14^, our SVM model shows better statistical performance in a model that deals with more samples in the test set.

## Conclusions

A three-descriptor SVM model with SVM parameters *C* of 7.2906 and *γ* of 1.7200 was successfully built for the skin permeability log *K*_p_ of a sufficiently large data set consisting of 274 compounds, by means of a genetic algorithm. The SVM model possesses *rms* errors of 0.253 for the training set (139 compounds), 0.302 for the test set (135 compounds), and 0.270 for the total set (274 compounds). Our SVM model shows better statistical performance in a model that deals with more samples in the test set, compared with other QSARs of the skin permeability of log *K*_p_ reported in the literature. There were non-linear relationships between the skin permeability log *K*_p_ and molecular descriptors used. It was reasonable applying a SVM algorithm to develop a nonlinear QSAR model for skin permeability.

## Supplementary Information

Supplementary Table S1.

## Data Availability

All data generated or analysed during this study are included in this published article (and its “Supplementary Information” files).
